# Osteoarticular Changes in Acromegaly

**DOI:** 10.1155/2012/839282

**Published:** 2012-09-12

**Authors:** Zdenko Killinger, Martin Kužma, Lenka Sterančáková, Juraj Payer

**Affiliations:** 5th Department of Internal Medicine, University Hospital, Medical Faculty of Comenius University, Ružinovská 6, 821 06 Bratislava, Slovakia

## Abstract

Acromegaly is caused by hypersecretion of growth hormone (GH) and consequently of insulin-like growth factor-I (IGF-1) due to pituitary tumor. Other causes, such as increased growth-hormone releasing hormone (GHRH) production, ectopic GHRH production, and ectopic GH secretion, are rare. Growth hormone and IGF-1 play a role in the regulation of bone metabolism, but accurate effect of growth hormone excess on bone is not fully explained. The issue of osteoarticular manifestations is still very actual, due to development of complications in the majority of patients with acromegaly. Traditionally, acromegaly is considered as a cause of secondary osteoporosis. Nowadays, it is discussed if BMD as predictor of osteoporotic fractures in acromegalic patient is decreased or even normal. Thus, bone quality remains to be more important in assessment of fracture risk. GH excess leads to increased bone turnover, defined by changes of bone markers. The articular manifestations are frequent clinical complications and may be present as the earliest symptom in a significant proportion of acromegalic patients. Articular manifestations are the main causes of morbidity and immobility of these patients, and they are persistent even after successful treatment. Quick recognition of osteoarticular changes and aiming the therapy lead to decrease in complication number.

## 1. Introduction

Acromegaly is a chronic endocrinopathy caused by hypersecretion of growth hormone (GH) and consequently of insulin-like growth factor-I (IGF-1) due to pituitary tumor. Other causes, such as increased growth hormone-releasing hormone production from hypothalamic tumors, ectopic growth hormone-releasing hormone production, and ectopic GH secretion from nonendocrine tumors, are rare. Growth hormone (GH) and its peripheral mediator, insulin-like growth factor-1 (IGF-1), play a significant role in the regulation of bone metabolism. While adult growth hormone deficiency has been shown to be involved in determining bone loss and osteoporosis, the effects of growth hormone excess on bone are unexplained and uncertain. It remains unclear whether the overall increase in BMD observed in patients with acromegaly is associated with a decrease in fracture risk [1]. Musculoskeletal pain is a frequent problem encountered in acromegaly and is associated with a reduction in quality of life. Joint symptoms are the most frequent complaint affecting approximately 70% of individuals at the time of diagnosis. Among musculoskeletal symptoms the most prevalent are arthropathy, carpal tunnel syndrome, proximal myopathy, and fibromyalgia. Musculoskeletal manifestations of acromegaly are frequent, and virtually all patients develop symptoms or signs related to arthropathy, but these signs have been poorly studied. The arthropathy in acromegaly can affect both axial and peripheral joints, and it may be present as the earliest clinical symptom of the disease. The most commonly involved joints are hips, shoulders, knees, hands, and elbows [2]. Early diagnosis and proper treatment of the diseases can prevent the development of irreversible complications of the disease and improve the quality of life in patients suffering from the disease. 

## 2. Effects of Growth Hormone on Bone and Joints

The anabolic actions of GH on many organ systems are well documented. During the childhood GH stimulates longitudinal bone growth. During the adolescence and early adulthood GH stimulates skeletal maturation till the achievement of peak bone mass-maximal bone mass, which is the main predictor of osteoporotic fracture risk. In adult age GH is important in the maintenance of bone mass through the regulation of bone turnover. Serum GH levels decline with increasing age (GH secretion reduces by approximately 14% for each decade of adult life after puberty and a dysfunctional GH axis may thus play a role in the pathogenesis of postmenopausal and senile osteoporosis [1, 3]). Growth hormone (GH) has an anabolic effect on bone *in vitro* and* in vivo.* GH and IGF-1 are important regulators of bone homeostasis through life. They are acting in autocrine and paracrine ways, stimulate proliferation, differentiation, and extracellular matrix production in osteoblastic like-cell lines and finally bone formation. GH also stimulates recruitment and bone resorption activity in osteoclastic-like cells [1].

The pathogenesis of arthropathy is complex, including both GH/IGF-1 excess and secondary degenerative changes. Based on experimental evidence, the pathophysiology of the acromegalic arthropathy can be predicted. At the inicial stage, GH excess stimulates local production of IGF-1 in cartilage which in connections with elevated levels of circulating IGF-1 results in replication and hyperfunction of articular chondrocytes and increased matrix synthesis. The cartilage begins to thicken, leading to widening of the joint, and hypermobility of joints. GH also stimulates connective cell hyperfunction, resulting in growth of periarticular structures, synovial hypertrophy further exacerbates the abnormal mechanical loading of the joints. In this phase arthropathy may be inverted by control of GH and IGF-1 hypersecretion. With disease progression, fissures develop on to the cartilage surface and progressively enlarge, whereas regenerative fibrocartilage proliferates disproportionately more than in osteoarthritis, presumably as a result of growth hormone stimulation. The regenerative fibrocartilage frequently becomes calcified, resulting in osteophyte formation. In advanced cases, fissures extended to the subchondral bone, widen, and become undercut, producing ulceration of the joint cartilage. The underlying bone shows an accelerated turnover, eburnation, and subchondral cyst formation. Ultimately, the articular cartilage becomes thinned with narrowing of the joint space, a process that hares many features with osteoarthritis. At this phase of acromegalic arthropathy can not be further improved by GH and IGF-1 suppression [4].

## 3. Epidemiology and Pathogenesis

Osteoarticular manifestations occur in the majority of patients with acromegaly. The delay between the estimated onset of acromegaly and the appearance of joint disease is approximately 10 years, but the range is wide. Early signs of joint involvement have also been reported in patients with short duration of the disease [4]. Radiographic changes in peripheral joints are widely, being found in more than 50% patients [5]. The articular manifestations of acromegaly are one of the most frequent clinical complications and may be present as the earliest symptom in a significant proportion of patients. Joint manifestation of acromegaly could even lead to decreased quality of life. It was described by Biermasz at al. [6] that the presence of joint-related complaints has highly significant impact on quality of life with reduced scores especially for the physical and general subscales and less pronounced impact for the mental subscales.

Less discussed but frequent sign is bone mass alteration leading to osteoporosis. Epidemiology of osteoporotic fractures is not enough reviewed and suggestions are different. Few past studies were aimed to bone quality comparing trabecular and cortical bone content. Trabecular bone was reduced in acromegalic patients compared with controls in study by Ueland et al. [7]. Recent studies are supposing reduced bone mineral density (even osteoporotic values of BMD) regardless of activity of disease. Over 50 years 40% of patients are osteoporotic in study by Madiera et al. [8]. Another study [1] showed similar number of patients with decreased BMD. In available literature no study is present concerning quality of life in acromegalic patient associated to reduced bone mineral density or higher prevalence of osteoporotic fractures. 

## 4. Prognosis

Incidence of hypermobility and functional restriction of movement is likely to depend on the duration of the disease. Articular manifestations are the main causes of morbidity and immobility of these patients [9]. The bone enlargement that occurs with excess secretion of GH is not reversible with successful treatment. Painful arthropathy often persists despite biochemical control, and joint complaints are a major contributor to a perceived reduced quality of life despite long-term biochemical remission [10].

## 5. Bone Turnover in Acromegalic Patients

**Figure 1:**
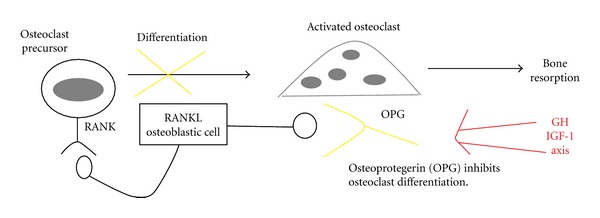
RANK-RANKL-osteoprotegerin mechanism of bone remodelation (adjusted by Lipincott Williams & Wilkins, South Med 2004).

It is well known that active acromegaly is associated with increased bone turnover markers. Bone turnover markers are peptides secreted by osteoblasts, osteoclasts, or by components of bone matrix, released in the circulation during bone resorption and formation. Several studies have shown a positive relationship between serum GH concentrations and markers of bone formation. The effect of chronically elevated levels of GH, respectively, IGF-1 on bone metabolism can be examined by determining the levels of markers of bone formation and resorption. Bone markers characterize the actual bone turnover of the skeleton. Osteomarkers help us determine the intensity of bone remodeling and respond very quickly to changes in bone metabolism, after 3–6 months. We could find possible analogy between acromegaly and treated GH deficiency. Replacement therapy with recombinant GH (rhGH) leads to increased bone turnover, which is defined by changes in biochemical markers of bone resorption and formation. The effect rhGH on bone remodeling is biphasic: rhGH causes a maximal effect on bone resorption after 3 months and on bone formation after 6 month. The effect on bone formation is sustained for prolonged periods of time. The effect of rhGH on biochemical markers of bone turnover is dose dependent but not influenced by the modality of administration. RhGH causes an increase in urinary and serum calcium after 3–6 months, an effect caused by calcium mobilization from the skeleton, an increase in intestinal calcium absorption and in the renal reabsorption of calcium due to increased sensitivity to PTH. RhGH is antiphosphaturic and increases the intestinal absorption of phosphate which leads to increased levels of serum phosphate. RhGH may also normalize the circadian rhythm of PTH secretion. Receptors for IGF and GH have been demonstrated in osteoclasts, thus GH and IGF-1 may directly affect their function and activity. In addition, GH/IGF-1 axis indirectly affects bone resorption by stimulating the release of paracrine mediators that regulate the resorption of bone. Critical for the bone resorptive process is the balance between the newly discovered members of the TNF ligand and receptor superfamilies, osteoprotegerin (OPG) and receptor activator of nuclear factor-kB ligand (RANKL). It is known that *in vitro* binding of RANKL to receptor RANK stimulates osteoclast differentiation, activates mature osteoclasts, and inhibits osteoclast apoptosis. OPG blocks the effects of RANKL by preventing binding to its receptor RANK (see Figure 1) [11]. Also another factor, osteoprotegerin (OPG), is involved in the regulation of osteoblasts and osteoclasts and in maintaining of bone mass. Recent study [12] has shown that treatment with growth hormone in patients with the deficiency was able to induce a significant increase in plasma OPG as well as cortical and trabecular bone. The result showed that exposure to growth hormone was able to stimulate OPG secretion in a concentration-dependent GH [12].

Ueland et al. [7] evaluated bone turnover in patients with acromegaly based on bone markers osteocalcin (formation) and CTx (resorption). Multivariate analysis identified age, serum IGF-1, and GH as independent predictors for CTx, while age and IGF-1 were independent determinants of osteocalcin. It was obvious that gonadal status did not affect the turnover parameters. Positive correlation between osteocalcin and CTx was observed in both genders, suggesting that bone turnover is synchronized. This synchronization is called “coupling phenomenon”. Coupling phenomenon was more discussed in studies with adult GH deficiency [13, 14], and it is defined as bone remodeling initiated by osteoclastic resorption, which is under physiological conditions temporarily followed by osteoblastic bone formation. Positive effect of GH on bone formation was confirmed by De Paula et al. in study with GH-deficient adults [13] where discontinuation of GH treatment resulted in a return of CTx levels to the baseline, but the effect on osteocalcin persisted despite discontinuation of treatment for at least 6 months. In our study with 94 growth hormone deficient adults [15] we have observed carboxy-terminal collagen crosslinks (CTX), marker of bone resorption, and it was increasing during first year of GH replacement treatment. After one year of treatment period CTX was slightly decreasing for 2 years. On the opposite site, levels of osteocalcin, marker of bone formation, were significantly rising during whole 2-year treatment period. This study demonstrated positive effect of GH on bone markers with predominance to bone formation. According to this study we could suppose that possible analogy exists with active acromegaly.

Parkinson et al. [16] evaluated the effect of pegvisomant to normalize biochemical parameters in 16 acromegalic patients. They found significantly higher levels of PIIINP (procollagen III N-terminal propeptide, marker of bone formation), CTx, and osteocalcin in patients at baseline. Pegvisomant induced normalization of serum IGF-1, which was associated with a significant decreased markers of bone formation and resorption. Decrease in the concentration of IGF-1 positively correlated with decreased levels of PIIINP. After normalization of IGF-1 there was no statistically significant difference between patients and controls for all parameters of bone turnover. Pegvisomant-induced normalization of IGF-1 is therefore associated with normalization of elevated bone turnover markers such as osteocalcin, CTx, and PIIINP. In contrast to other studies, this study has not observed significant correlation between osteocalcin and serum GH or IGF-1, probably due to small size of study cohorts. A significant positive correlation between GH and osteocalcin was observed in study of Piovesan et al. [17], where positive correlation between GH and osteocalcin was observed before administrating of octreotide as well in osteocalcin levels and IGF-1. Decrease in levels of GH, IGF-1, and osteocalcin was observed after treatment with octreotide. This study also demonstrated that patients with active acromegaly have increased levels of osteocalcin and PIIINP as the reflect of increased osteoblastic activity. 

To summarize, active acromegaly leads to increase in bone turnover markers. GH excess inhibits differentiation and activity of osteoclasts through the RANK-RANK-l—OPG system. Independent predictors of bone turnover markers are values of GH, IGF-I as well as age of the patient. Studies have proven correlation between bone resorption and formation, suggesting coupling between bone turnover markers. After treatment with somatostatin analogues (pegvisomant and octreotide) bone markers and IGF-I normalize. Study of transsphenoidal surgery effect on bone turnover is missing.

## 6. Bone Mineral Density in Acromegalic Patients

The whole effect of growth hormone excess on bone mineral density is not fully explained. GH/IGF-1 axis might play an important role in the maintenance of bone mass, what was described by few past studies [18]. It was confirmed that BMD of lumbar spine and femoral neck was increased by patients with active acromegaly. Most studies suggest that cortical bone mass is increased in acromegaly, whereas trabecular bone seems largely unaffected, confirming that the actions of GH are mediated also by local produced IGFs [19]. Recently, studies have proven lower BMD (even osteoporosis) in patients with active and controlled acromegaly. Madiera et al. [8] have observed *Z*-score below −2SD in almost 30% of study patients with acromegaly, and almost 40% patients over 50 years were osteoporotic. There was no difference in *Z*-score and *T*-score between groups with active or controlled acromegaly. Giuseppina et al. [1] have observed significantly reduced BMD in 42% of patients, where men showed lower *T*-score of femoral neck, regardless of gonadal status. Study by Madiera et al. [8] brought also other interesting findings such as comparison of gonadal status in acromegalic patients with active disease. Higher *T*-score was observed in eugonadal patients with active acromegaly compared to controlled hypogonadal patients and the most of osteoporotic patients were hypogonadal. The effect of gonadal status on BMD in acromegalic patients is ambiguous. Some studies [20, 21] have proven positive effect of eugonadism on bone mineral density, but some studies [22, 23] have shown controversial results, higher bone mineral density regardless of gonadal status. The most affected measured site was distal radius because the results of BMD of femur or lumbar spine could be distorted by periarticular calcifications and cartilage damage, which is very common in acromegaly [24]. Association between gonadal status and bone mineral density was found by univariate testing, but multivariate analysis has confirmed the importance of age and gender. Age and gender could by defined as main determinants of bone remodelation mechanisms, indicating that the same process influences bone loss in normal population, growth-hormone-deficient adults and also in acromegalic patients [7]. We have proven gender difference in influence of GH/IGF-1 axis on bone mass in growth hormone deficient adults after 2 years treatment with rhGH [15]. There was an increase in BMD of lumbar spine in men (15,8%) in comparison to women (5,6%). Similar results were confirmed in femoral neck region (men 11%, women 3%). According to published discrepancies between studies concerning BMD in acromegalic patients, BMD seems to be not a proper marker for fracture risk assessment. Adequately designed studies focusing on quality of bone in patient with acromegaly are missing, and new methods for bone quality assessment explaining fracture risk are required. Possibly beneficial seems to be volumetric measuring using CT, MRI, or trabecular bone structure (TBS) assessment using DXA technology. According to existing criteria of osteoporosis risk it is not possible to asses exact risk of fracture, similarly as it was described in patients suffering from diabetes mellitus type II. 

In conclusion, GH/IGF-1 axis plays clearly an important role in maintenance of bone mass. It was described that cortical bone in acromegaly changes in opposite to trabecular bone. Results of few past studies remain controversial. Recent studies suggest that BMD decreases in patients with active controlled disease. As it was described in previous text influence of gonadal status plays also the role in bone mass acquisition, proving that hypogonadal patients with controlled disease have higher prevalence of osteoporosis in comparison to eugonadal noncontrolled acromegalic patients. According to gonadal status also gender difference was described in acromegalic patients, supported by studies with GH deficient patients treated with recombinant human growth hormone. Less discussed, but very important, was BMD measurement technique in past studies. It suggested that two-dimensional DXA is distorted because it is not counting with perpendicular scan bone density. Thus, other techniques should be used for assessment of BMD. At last, the clinical access to acromegalic patient should be individual with emphasis to state of disease, gonadal status, gender, age, measured site of bone measuring technique.

## 7. Fracture Risk in Acromegalic Patients

Only in one study [25] increased prevalence of radiological vertebral fractures in postmenopausal women with acromegaly was observed. It is unknown whether increased fracture risk is a result of different risk factors in this population. As it was mentioned, acromegaly is traditionally considered as a cause of secondary osteoporosis, but BMD is not decreased and its measurement is overestimated because of structural modification of spine. In another studies, it was shown that GH excess has effect on trabecular bone but no effect on cortical bone [4, 26]. Lower bone mass of trabecular bone could be influenced by many factors, but the most probable seems to be hypogonadism. Circulating sex hormones have better affinity to trabecular bone and in spite of sex hormones low levels in this group of patients are able to affect trabecular bone. Interesting finding was published in recent study of Giuseppina et al. [1] where vertebral fractures were observed even in patients with normal BMD (g/cm^2^) regardless of gender. Based on these findings an insufficient quality of bone should be the most important factor of bone health, and it is influenced by gonadal status, disease activity, and gender. Full-understanding of the problematic of osteoporotic fractures in patients with acromegaly requires other studies with higher number of patients.

## 8. Arthropathy in Acromegalic Patients

**Figure 2:**
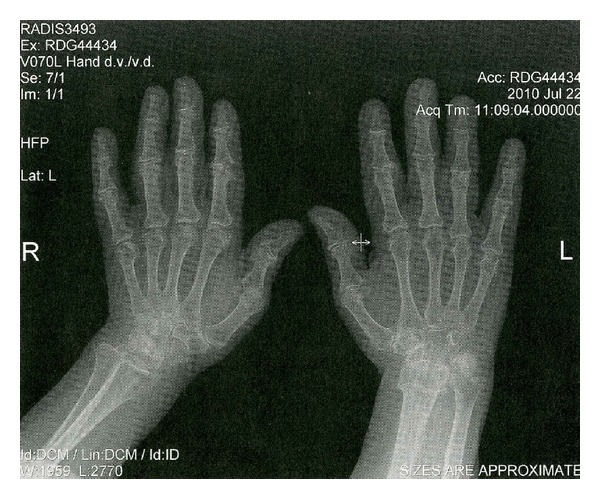
Radiological changes in acromegalic arthropathy on X-ray scan (with permission of Peter Vaňuga, MD, PhD., National institute of endocrinology and diabetology, L'ubochňa, Slovakia).

The articular manifestations of acromegaly are one of the most frequent clinical complications and may be present as the earliest symptom of acromegaly. Its prevalence and severity worsen with the duration of uncontrolled disease and often result in significant disability. The pathogenesis of arthropathy in acromegaly is comprised of two mechanisms: initial endocrine and subsequent mechanical changes [27]. Radiological changes in this early phase are joint space widening and periarticular soft tissue hypertrophy. With ongoing disease arthropathy becomes irreversible and biochemical control of acromegaly, as documented by a normal IGF-I, will have a very small efficacy in improving the clinical status. Altered joint geometry results in repeated intraarticular trauma and exaggerated a reparative reaction which leads to scar, cysts, and osteophyte formation with further worsening of joint geometry. At this point, the disease acquires the characteristics and features of degenerative joint disease [28]. Radiographic changes at this stage are characterized by narrowing of joint spaces (see Figure 2), osteophytosis, cysts, and other features typical for the later stages of the disease (see Table 1) [29]. 

The radiological appearance of arthropathy in acromegaly was mostly studied in small noncontrolled groups of patients with untreated and treated but active disease. These studies have suggested that more severe radiological abnormalities were related to biochemically more active acromegaly and longer disease duration [30]. 

Further common complaints relate to limited range of movement, joint instability, and joint deformation. The presence of radiologic abnormalities and clinical manifestations of arthropathy are not correlated, unless joints are severely affected as in long-standing disease [28].

There were few studies evaluating prevalence of joint changes in acromegalic patients. Recent study evaluated 89 acromegalic patients with adequate long-term disease control for prevalence and radiological characteristics of arthropathy. They found evidence for radiological arthritis in a least one joint in all patients and clinical arthritis in two-thirds of patients. The most prevalent manifestation was axial osteoarthritis, affecting the cervical and lumbar areas, even at young ages. The characteristic radiological changes observed were wide joint spaces and severe osteophytosis [3].

In early phase of the disease widened intervertebral spaces and vertebral enlargement may be present in the spine X-ray. Ossification of the anterior surface of vertebral bodies is relatively common and in more severe cases can bridge the disc space resembling diffuse idiopathic skeletal hyperostosis syndrome. Biermasz et al. [6, 31] reported that a high prevalence of self-reported joint complaints persisted despite successful long-term treatment of acromegaly. These joint problems were an important indicator of impaired quality of life. 

## 9. Summary

Osteoarticular manifestations of acromegaly are the most frequent clinical complications and may be present as the earliest symptom in a significant proportion of patients with acromegaly. Many patients with joint complaints are misdiagnosed as a generalized osteoarthritis. Early diagnosis of acromegalic arthropathy and aiming the therapy could lead to decrease in severe joint complications, a disability. With ongoing disease arthropathy becomes irreversible and biochemical control of acromegaly, as documented by a normal IGF-I, will have a very small efficacy in improving the clinical status.

The effects of growth hormone excess on bone turnover and bone density are not fully explained, but GH/IGF-1 axis plays an important role in the maintenance of bone mass. This effect is proved by high levels of bone turnover markers in active acromegaly, and correlation between IGF-I and bone remodeling was proven by few past studies. Traditionally, acromegaly is considered as a cause of secondary osteoporosis. Nowadays, it is frequently discussed if BMD as predictor of osteoporotic fractures in patient with acromegaly is unchanged according to earlier studies or decreased according to few recent studies. It was shown that GH excess has effect on trabecular bone but no effect on cortical bone, thus quality of bone remains to be more important in osteoporotic fracture risk, but another studies are needed. Supposed increased fracture risk cannot be fully explained by changes in bone mineral density. It seems that bone quality plays the most important role in fracture risk regardless of BMD. Nowadays, in clinical practice noninvasive methods to asses bone quality are missing. Adequately designed studies focusing on different parameters of bone quality in patient with acromegaly are required. Assessment of patient risk profile could be helpful in stratification of patients with high risk of fracture. In clinical practice beside BMD testing and bone turnover evaluation fracture risk assessment using FRAX calculator could be helpful in fracture risk prediction. Prevalence and severity of arthropathy worsen with the duration of uncontrolled disease and often result in significant disability. Studies have suggested more severe joint abnormalities related to biochemically more active and longer duration of acromegaly thus quick obtaining of diagnosis and aiming the therapy lead to decrease in fatal complication number.

## Figures and Tables

**Table 1:** Review of radiological findings in acromegaly [27].

Radiologic findings in acromegalic joint disease
Increased joint space diameter
Decreased joint space diameter (severe disease)
Tufting of distal phalanges
Enthesopathy
Angular joint deformities
Osteophyte formation
Articular surface calcification
Eburnation
Subchondral cyst formation
Costochondral joint calcification and enlargement
Vertebral body enlargement
